# Corticosteroid treatment modulates immune cell populations in the glioblastoma microenvironment

**DOI:** 10.1093/noajnl/vdag258

**Published:** 2026-10-07

**Authors:** Rune Just Clausen, Sofie Sorgenfri Hjerrild Nielsen, Julie Jacobsen, Line Viftrup Buchwald, Vilde Pedersen, Nadine Margaretha Hammouda, Nicolai Schou Bager, Kirsten Grunnet, Hans Skovgaard Poulsen, Simone Maarup, Benedikte Hasselbalch, Thomas Urup, Signe Regner Michaelsen, Sara Blaabjerg Artzi, Bjarne Winther Kristensen

**Affiliations:** Department of Pathology, The Bartholin Institute, Rigshospitalet, Copenhagen University Hospital, Copenhagen, Denmark; Department of Clinical Medicine and Biotech Research & Innovation Centre (BRIC), University of Copenhagen, Copenhagen, Denmark; DCCC Brain Tumor Center, Rigshospitalet, Copenhagen University Hospital, Copenhagen, Denmark; Department of Pathology, The Bartholin Institute, Rigshospitalet, Copenhagen University Hospital, Copenhagen, Denmark; Department of Clinical Medicine and Biotech Research & Innovation Centre (BRIC), University of Copenhagen, Copenhagen, Denmark; DCCC Brain Tumor Center, Rigshospitalet, Copenhagen University Hospital, Copenhagen, Denmark; Department of Pathology, The Bartholin Institute, Rigshospitalet, Copenhagen University Hospital, Copenhagen, Denmark; Department of Clinical Medicine and Biotech Research & Innovation Centre (BRIC), University of Copenhagen, Copenhagen, Denmark; DCCC Brain Tumor Center, Rigshospitalet, Copenhagen University Hospital, Copenhagen, Denmark; Department of Pathology, The Bartholin Institute, Rigshospitalet, Copenhagen University Hospital, Copenhagen, Denmark; Department of Clinical Medicine and Biotech Research & Innovation Centre (BRIC), University of Copenhagen, Copenhagen, Denmark; DCCC Brain Tumor Center, Rigshospitalet, Copenhagen University Hospital, Copenhagen, Denmark; Department of Pathology, The Bartholin Institute, Rigshospitalet, Copenhagen University Hospital, Copenhagen, Denmark; Department of Clinical Medicine and Biotech Research & Innovation Centre (BRIC), University of Copenhagen, Copenhagen, Denmark; DCCC Brain Tumor Center, Rigshospitalet, Copenhagen University Hospital, Copenhagen, Denmark; Department of Pathology, The Bartholin Institute, Rigshospitalet, Copenhagen University Hospital, Copenhagen, Denmark; Department of Clinical Medicine and Biotech Research & Innovation Centre (BRIC), University of Copenhagen, Copenhagen, Denmark; DCCC Brain Tumor Center, Rigshospitalet, Copenhagen University Hospital, Copenhagen, Denmark; Department of Pathology, The Bartholin Institute, Rigshospitalet, Copenhagen University Hospital, Copenhagen, Denmark; Department of Clinical Medicine and Biotech Research & Innovation Centre (BRIC), University of Copenhagen, Copenhagen, Denmark; DCCC Brain Tumor Center, Rigshospitalet, Copenhagen University Hospital, Copenhagen, Denmark; DCCC Brain Tumor Center, Rigshospitalet, Copenhagen University Hospital, Copenhagen, Denmark; Department of Oncology, Rigshospitalet, Copenhagen University Hospital, Copenhagen, Denmark; DCCC Brain Tumor Center, Rigshospitalet, Copenhagen University Hospital, Copenhagen, Denmark; Department of Oncology, Rigshospitalet, Copenhagen University Hospital, Copenhagen, Denmark; DCCC Brain Tumor Center, Rigshospitalet, Copenhagen University Hospital, Copenhagen, Denmark; Department of Oncology, Rigshospitalet, Copenhagen University Hospital, Copenhagen, Denmark; DCCC Brain Tumor Center, Rigshospitalet, Copenhagen University Hospital, Copenhagen, Denmark; Department of Oncology, Rigshospitalet, Copenhagen University Hospital, Copenhagen, Denmark; DCCC Brain Tumor Center, Rigshospitalet, Copenhagen University Hospital, Copenhagen, Denmark; Department of Oncology, Rigshospitalet, Copenhagen University Hospital, Copenhagen, Denmark; Department of Pathology, The Bartholin Institute, Rigshospitalet, Copenhagen University Hospital, Copenhagen, Denmark; Department of Clinical Medicine and Biotech Research & Innovation Centre (BRIC), University of Copenhagen, Copenhagen, Denmark; DCCC Brain Tumor Center, Rigshospitalet, Copenhagen University Hospital, Copenhagen, Denmark; Department of Pathology, The Bartholin Institute, Rigshospitalet, Copenhagen University Hospital, Copenhagen, Denmark; Department of Clinical Medicine and Biotech Research & Innovation Centre (BRIC), University of Copenhagen, Copenhagen, Denmark; DCCC Brain Tumor Center, Rigshospitalet, Copenhagen University Hospital, Copenhagen, Denmark; Department of Pathology, The Bartholin Institute, Rigshospitalet, Copenhagen University Hospital, Copenhagen, Denmark; Department of Clinical Medicine and Biotech Research & Innovation Centre (BRIC), University of Copenhagen, Copenhagen, Denmark; DCCC Brain Tumor Center, Rigshospitalet, Copenhagen University Hospital, Copenhagen, Denmark

**Keywords:** corticosteroids, digital pathology, glioblastoma, immune microenvironment, prednisolone

## Abstract

**Background:**

Corticosteroids play a crucial role in alleviating tumor-associated edema in patients with brain tumors. However, their impact on the tumor microenvironment within glioblastoma remains largely unexplored. In the context of increasing interest in immunotherapy and limited clinical progress for glioblastoma patients, it is important to understand how corticosteroid therapy influences the immune microenvironment.

**Methods:**

This study involved 80 glioblastoma patients, 41 of whom received corticosteroids preoperatively and 39 who did not. The groups were balanced for key clinical prognostic parameters. Through immunohistochemical staining and image analysis-based software classifiers, we quantitatively assessed a broad marker panel covering immune, myeloid, hypoxia-related, and tumor-associated features of the glioblastoma microenvironment.

**Results:**

Our analysis revealed a more than 2-fold decrease in FOXP3+ regulatory T cells in glioblastoma tissue samples from patients who received corticosteroids. In dose-subgroup analyses, high-dose corticosteroid exposure was associated with increased CD68+ and CD204+ myeloid/macrophage-marker abundance. CD8 and CD4 T cell counts, as well as CD86, TMEM119, HIF-1α, PD-L1, and SOX2, remained similar across all groups.

**Conclusions:**

Our findings suggest that corticosteroid treatment is associated with selective changes in T cell and myeloid/macrophage-marker abundance within the tumor microenvironment. These findings highlight corticosteroid exposure as an important factor to consider when interpreting immune profiles and developing immune-based therapies in glioblastoma.

Key Points

- Corticosteroids are associated with fewer FOXP3+ regulatory T cells in GBM.
- High-dose corticosteroids are associated with increased CD68+ and CD204+ myeloid/macrophage-marker abundance.
- Corticosteroid use should be considered in immune profiling studies of GBM.

Importance of the Study

Corticosteroids are a cornerstone of symptomatic management in glioblastoma, yet their direct effects on the tumor microenvironment are poorly defined and often overlooked in clinical studies. Here, we show that preoperative corticosteroid therapy is associated with a substantial reduction in FOXP3+ regulatory T cells and a high-dose-associated increase in CD68+ and CD204+ myeloid/macrophage-marker abundance in human glioblastoma tissue. These findings suggest that corticosteroid exposure selectively alters the intratumoral immune landscape, extending beyond its well-known systemic immunosuppressive effects.

The high-dose-associated myeloid/macrophage-marker pattern may have implications for immune-based therapies and could contribute to variability in immune profiling and treatment response. Importantly, our results identify corticosteroid exposure as an important and often underappreciated factor that should be considered in translational and clinical studies, with implications for patient stratification, biomarker interpretation, and trial design.

The symptoms of glioblastoma (GBM) vary depending on the tumor’s location, size, and growth rate. Common early signs include epileptic seizures, headaches, and dizziness.^1^ These symptoms often arise from significant edema in the brain tissue surrounding the tumor.^2^ In the preoperative setting, corticosteroid treatment, like dexamethasone or methylprednisolone, is frequently used to manage this edema.^3^^,^^4^ Typically, patients receive substantial intravenous doses, ranging from 10 to 20 mg/day of dexamethasone or 50 to 100 mg/day of methylprednisolone.^4^ Although corticosteroids are well known for their anti-inflammatory and immunosuppressive effects, their impact on the GBM immune microenvironment remains unclear.^5^ Clinical studies using peripheral blood immune profiling by flow cytometry have shown systemic immunosuppression associated with reduced survival in GBM patients,^6-8^ including marked decreases in circulating immune cells such as γδ T cells and NK cells.^6^ However, it remains uncertain whether these effects extend to the tumor microenvironment (TME).

Although corticosteroid treatment has been demonstrated to alter circulating myeloid cell counts in GBM,^6^ it remains unclear whether corticosteroid treatment influences tumor-associated microglia/macrophages (TAMs) in the TME of GBMs. TAMs, which consist of both brain-resident microglia and recruited monocyte-derived macrophages, not only play critical roles in shaping the TME and promoting GBM progression,^9^ but also exert potent immunosuppressive effects through cytokine secretion, checkpoint expression, and recruitment of regulatory T cells.^10^ One study compared dexamethasone-treated and untreated GBM samples using immunohistochemical staining for CD163 and found that dexamethasone significantly decreased the infiltration of CD163+ TAMs in the TME.^11^ However, this finding was based on a cohort of eight patients in each group, and it has not yet been validated in studies with well-matched clinical parameters between patient groups.

Most studies investigating the effects of corticosteroid treatment on immune cells are based on experimental cell and animal models, in addition to a few flow-cytometric studies on human blood samples.^12^ Findings from animal models, however, have shown varying results. For example, a rat glioma model showed that dexamethasone significantly reduced microglia and lymphocyte infiltration in brain tumors, while macrophage infiltration was unaffected.^13^ In a murine GBM model, dexamethasone was found to reduce the infiltration of myeloid and lymphoid cells in tumors by inhibiting interleukin-1 production and signaling in TAMs.^14^ Similarly, another study in an immunocompetent murine GBM model demonstrated that dexamethasone significantly reduced intratumoral myeloid cell populations and T cells, including CD4+, CD8+, and regulatory subsets.^15^

We have previously shown that neoadjuvant PD-1 blockade in patients with recurrent GBM failed to induce consistent transcriptional changes in either tumor cells or tumor-associated macrophages, which likely reflects the overall scarcity of T cells in these tumors.^16^ In the context of the sustained interest in immunotherapy in brain cancer, a better understanding of how corticosteroids shape the immune landscape of GBM is therefore essential, as corticosteroid-associated changes in T cell and myeloid populations may influence how immune biomarkers and treatment responses are interpreted.

One central component of this immune landscape is immune checkpoint regulation. The immunosuppressive microenvironment of GBM is in part maintained by programmed death ligand 1 (PD-L1), a transmembrane protein expressed by multiple cell types in the TME, including tumor cells, T cells, and TAMs.^17-19^ PD-L1 binds to programmed cell death protein 1 (PD-1), a coinhibitory receptor expressed especially on activated T cells, thereby inhibiting T cell-mediated anti-tumor activity.^20-22^

The effect of corticosteroids on PD-L1 expression in GBM remains unclear. Experimental studies have reported inconsistent effects of corticosteroids on PD-L1, ranging from upregulation to downregulation depending on the model.^23-25^ Conversely, a clinical study in non-small cell lung cancer did not find a clear association between corticosteroid treatment and PD-L1 expression.^26^ Together, these findings indicate that the impact of corticosteroids on immune regulation in GBM is still unresolved.

In summary, while corticosteroids remain the drug of choice for managing GBM-related edema, their broader impact on the TME remains unclear. In this study, we used quantitative immunohistochemistry to examine how preoperative corticosteroid exposure is associated with T cell, myeloid/macrophage, immune checkpoint, hypoxia-related, and tumor-associated marker patterns in primary *IDH*-wildtype GBM tissue. These analyses were designed to clarify whether corticosteroid use is associated with local immune changes that may influence biomarker interpretation and immune-based therapeutic strategies.

## Methods

### Patient Tissue Inclusion

This study included formalin-fixed paraffin-embedded (FFPE) tissue samples from GBM patients who underwent surgery at Copenhagen University Hospital, Denmark, from 2012 to 2022. Eligible samples of tumor tissue were from patients with primary *IDH*-wildtype GBM. Furthermore, all patients included received standard-of-care treatment with radiation and temozolomide according to the Stupp protocol following primary surgery.

Patients who received >10 mg prednisolone per day preoperatively were defined as corticosteroid-treated and were compared with patients who did not receive preoperative corticosteroids. Patients were identified using an institutional GBM database at Copenhagen University Hospital. First, patients not using preoperative corticosteroids were identified. Then, corticosteroid-treated patients were selected by matching well-known independent prognostic factors such as age, ECOG/WHO performance status (PS), O6-methylguanine-DNA methyltransferase (*MGMT*) promoter methylation status, and multifocal disease. MRI-based tumor size was assessed on preoperative imaging and expressed as tumor area in mm^2^. All included patients were registered as *IDH*-wildtype, based on immunohistochemical assessment or next-generation sequencing.

FFPE tissue from primary GBM patients was considered for inclusion if it contained sufficient material (at least five mm in diameter) of acceptable quality and was representative of GBM. For each tumor, one representative FFPE tissue block was selected, and one whole tissue section from this block was analyzed for each immunohistochemical marker. Tissue slides were examined for GBM characteristics, with a primary focus on high cellularity, to ensure accurate representation of tumor tissue. The assessment further aimed to confirm the absence of significant necrosis, hemorrhage, and diffusely infiltrated brain parenchyma. All tissue sections were reviewed by a neuropathologist (B.W.K.).

### Immunohistochemistry

The primary antibodies used in this study are detailed in Table S1, and a summary of each antibody’s function is provided in Table S2.

Sections (3 μm thick) were cut from the FFPE tissue blocks, mounted on glass slides, and deparaffinized. Sections intended for immunohistochemistry underwent heat-induced epitope retrieval (HIER) followed by blocking of endogenous peroxidase activity using 3% H2O2 inhibitor and incubation with specific antibodies against SOX2 (Invitrogen/eBioscience), CD68 (Roche), CD204 (Cosmo Bio Co., Ltd.), CD4 (Roche), CD8 (Dako), FOXP3 (Invitrogen), PD-L1 (Agilent), CD86 (R&D Systems), TMEM119 (Abcam), and HIF-1α (BD Transduction Laboratories). Immunohistochemical staining procedures were conducted on the Discovery Ultra platform (Ventana Medical Systems). Antibody detection was carried out using the Omnimap detection system, and immunoreactivity was visualized using the ChromoMap DAB kit, followed by counterstaining with Hematoxylin II and Bluing Reagent. Separate sections were stained with hematoxylin and eosin (H&E) using a standard staining protocol. A tissue multiblock, comprising tissue from human colon, tonsil, placenta, cerebellum, and nine different GBMs, served as both negative and positive controls during staining optimization.

### Digital Image Acquisition and Automated Quantitative Analysis

H&E and immunohistochemically stained slides were digitized using NanoZoomer whole-slide scanners (NanoZoomer 2.0-HT and NanoZoomer S360; Hamamatsu Photonics, Hamamatsu, Japan) with a 40x objective. Image analyses were conducted using the Visiopharm Integrator System (VIS) image analysis software (version 2023.01 and version 2024.07 for analysis of PD-L1 expression). Within the image analysis module of VIS, all digitized images were manually delineated with regions of interest (ROIs), focusing on predominantly viable tumor tissue with high cellular density. Areas containing extensive necrosis, hemorrhage, vessels, blurring, folds, or other tissue artifacts were excluded. Smaller necrotic foci and surrounding pseudopalisading tumor cells were included when present within otherwise viable tumor tissue.

The following markers were analyzed: CD8, CD4, FOXP3, CD68, CD204, CD86, TMEM119, HIF-1α, SOX2, and PD-L1. For each marker, application protocols (APPs) were developed in the VIS software, using an intensity threshold classification system. Threshold settings were guided by the intensity of the DAB signal and refined in consultation with neuropathologist B.W.K. to ensure accurate identification of true positive staining. To identify marker-positive cells and ensure robust cell recognition, nuclei were first detected and segmented using a nuclei detection APP based on artificial intelligence (AI). The results of the automated image analysis within the designated ROIs were exported as either area fractions (DAB-positive area in μm^2^/total ROI area in μm^2^) or counts per area (DAB-positive cell count/total ROI area in μm^2^). Area fractions were subsequently converted to percentages, and counts per area were converted to counts/mm^2^. For HIF-1α, nuclear staining was quantified as the percentage of positive nuclei among all detected nuclei.

For CD86, 5 corticosteroid-treated patients were excluded because tissue was unavailable or staining could not be evaluated.

### Statistical Analysis

All statistical analyses and figure generation were performed in R. Baseline clinical characteristics were compared between patients who received preoperative corticosteroids and patients who did not. Continuous variables were compared using Wilcoxon rank-sum tests, and categorical variables were compared using Fisher’s exact tests. *MGMT* promoter methylation status was compared among cases with known methylation status.

Because marker distributions were generally skewed, marker abundances were summarized using medians and analyzed using non-parametric tests. For comparisons between patients with and without preoperative corticosteroid treatment, differences were evaluated using Wilcoxon rank-sum tests. For corticosteroid dose subgroup analyses, differences across the three groups were evaluated using Kruskal-Wallis tests, followed by Dunn’s post hoc tests for pairwise comparisons.

Spearman correlation analysis was used to assess pairwise relationships between marker abundances. Spearman correlation analysis was also used to assess the relationship between tumor size and selected markers. To examine whether tumor size influenced the corticosteroid-associated marker changes, rank-based linear regression models were fitted with marker rank as the outcome. Models included tumor size together with either corticosteroid exposure status or corticosteroid dose group. Tumor size was scaled per 1000 mm^2^ in these models.

Follow-up for overall survival was updated through June 15, 2026. Overall survival was evaluated using Kaplan-Meier plots, and statistical significance was determined using the Mantel-Cox log-rank test. *P* values < .05 were considered statistically significant. Significance levels are denoted with asterisks as follows: *P < .05, **P < .01, ***P < .001, and ****P < .0001.

## Results

### Cohort and Study Design

We analyzed preoperative primary tumor samples from 80 patients with *IDH*-wildtype GBM. Of these, 41 patients received corticosteroids before surgery and 39 did not. The two groups were well balanced in terms of age, sex, PS, tumor location, multifocal disease, and *MGMT* promoter methylation status (Table 1). We also noted that MRI-based tumor size was larger in patients who received corticosteroids, consistent with corticosteroid use being more frequent in patients with greater tumor burden (Table 1 and Figure S1A and B).

**Table 1.** Summary of the characteristics of included patients

Characteristic	Total (*N* = 80)	0 mg corticosteroid (*N* = 39)	12.5-120 mg corticosteroid (*N* = 41)	*P*-value
**Age (years)**				.396
Age > 60, no. (%)	32 (40)	15 (38)	17 (41)	.823
Median	58	57	59	
Range	29-76	29-76	39-73	
**Sex, no. (%)**				.061
Female	27 (34)	9 (23)	18 (44)	
Male	53 (66)	30 (77)	23 (56)	
**WHO performance status, no. (%)**				1.000
0	55 (69)	27 (69)	28 (68)	
1	20 (25)	10 (26)	10 (24)	
2	5 (6)	2 (5)	3 (7)	
**Location, no. (%)**				.161
Temporal	26 (32)	13 (33)	13 (32)	
Frontal	18 (22)	12 (31)	6 (15)	
Other	36 (45)	14 (36)	22 (54)	
**Corticosteroid dose (mg/day)**				N/A
Average	N/A	0	86.8	
**Multifocal disease at diagnosis, no. (%)**				.350
Yes	12 (15)	4 (10)	8 (20)	
No	68 (85)	35 (90)	33 (80)	
**Tumor size on preoperative imaging (mm^2^)**				<.001
Mean	1281	888	1557	
**MGMT promoter status, no. (%)**				.096
Methylated	28 (35)	9 (23)	19 (46)	
Unmethylated	47 (59)	25 (64)	22 (54)	
Missing	5 (6)	5 (13)	0 (0)	

*P*-values compare patients who received preoperative corticosteroids with patients who did not.

Continuous variables were compared using Wilcoxon rank-sum tests, and categorical variables were compared using Fisher’s exact tests.

### Quantitative Image Analysis of the TME

To assess how corticosteroid exposure relates to the TME, we performed immunohistochemical staining for CD8, CD4, FOXP3, CD68, CD204, CD86, TMEM119, HIF-1α, SOX2, and PD-L1 (Tables S1 and S2), and used a supervised image analysis pipeline to identify and quantify positive cells, positive area, or positive nuclei, depending on the staining pattern (Figures 1 and 2; Methods). These markers were selected to capture major immune and tumor-associated features of the GBM microenvironment, including T cell populations (CD8, CD4, FOXP3), tumor-associated microglia/macrophages and myeloid activation (CD68, CD204, CD86), microglia-associated signal (TMEM119), hypoxia-related changes (HIF-1α), tumor cells (SOX2), and immune checkpoint expression (PD-L1) (Table S2).

**Figure 1.**
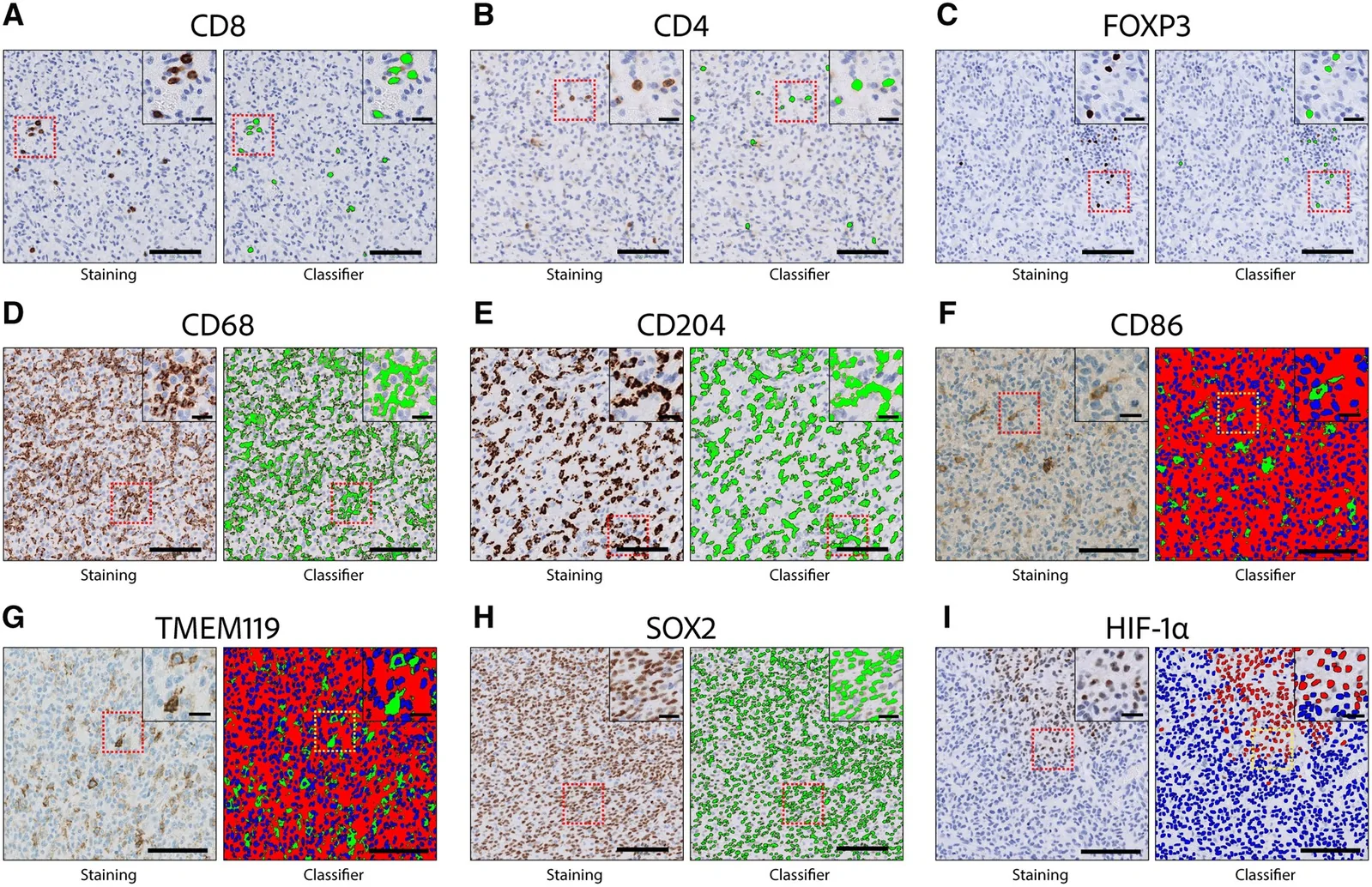
Representative immunohistochemical staining and automated image analysis. (A) CD8; (B) CD4; (C) FOXP3; (D) CD68; (E) CD204; (F) CD86; (G) TMEM119; (H) SOX2; (I) HIF-1α. For each marker, the left image shows representative immunohistochemical staining and the right image shows the corresponding Visiopharm analysis with classifier overlay. Dashed boxes indicate the regions shown at higher magnification in the insets. Scale bars are 100 μm in the main images and 20 μm in the insets.

**Figure 2.**
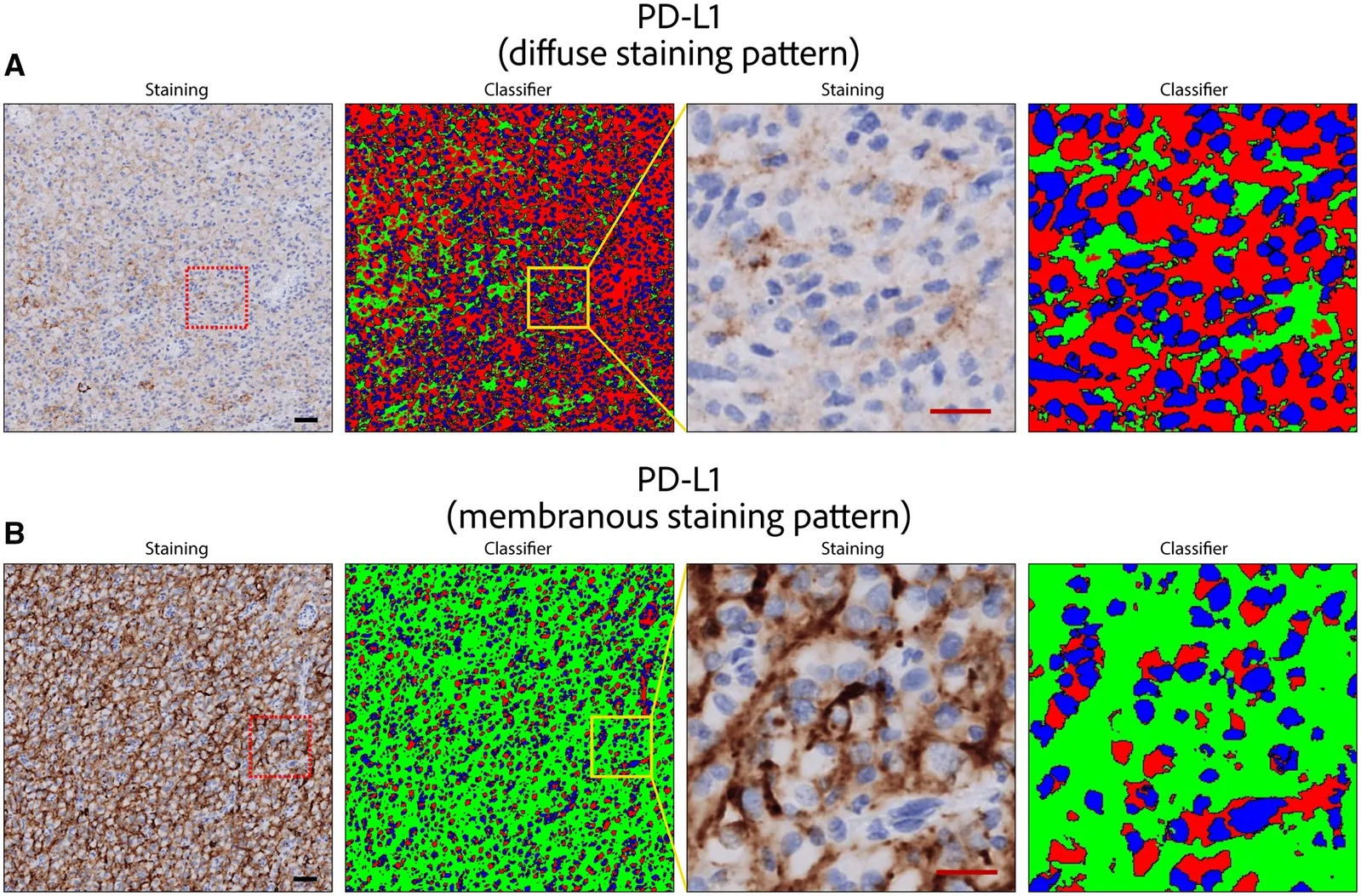
Representative PD-L1 staining patterns and automated image analysis. (A) Diffuse PD-L1 staining pattern. (B) Membranous PD-L1 staining pattern. Representative PD-L1 immunohistochemical staining images and corresponding Visiopharm analysis examples with classifier overlays are shown. Black scale bars represent 50 μm and red scale bars represent 25 μm. PD-L1 staining showed variable positivity patterns, with diffuse staining predominating. A more membranous, specific, and often more intense staining pattern was also observed, either alongside diffuse staining or in tumors with only minor diffuse positivity.

### Corticosteroid Exposure Is Associated With Shifts in T-Cell and Myeloid/Macrophage-Marker Abundance

We first compared marker abundance between patients who did and did not receive preoperative corticosteroids (Figure 3A-J, Table S3). FOXP3+ regulatory T cells were significantly reduced in corticosteroid-treated tumors, with a median of 1.19 FOXP3+ cells/mm^2^ compared with 2.53 cells/mm^2^ in non-treated tumors (*P* = .021). In contrast, no significant differences were observed for the remaining markers, including CD8, CD4, CD68, CD204, CD86, TMEM119, HIF-1α, SOX2, and PD-L1.

**Figure 3.**
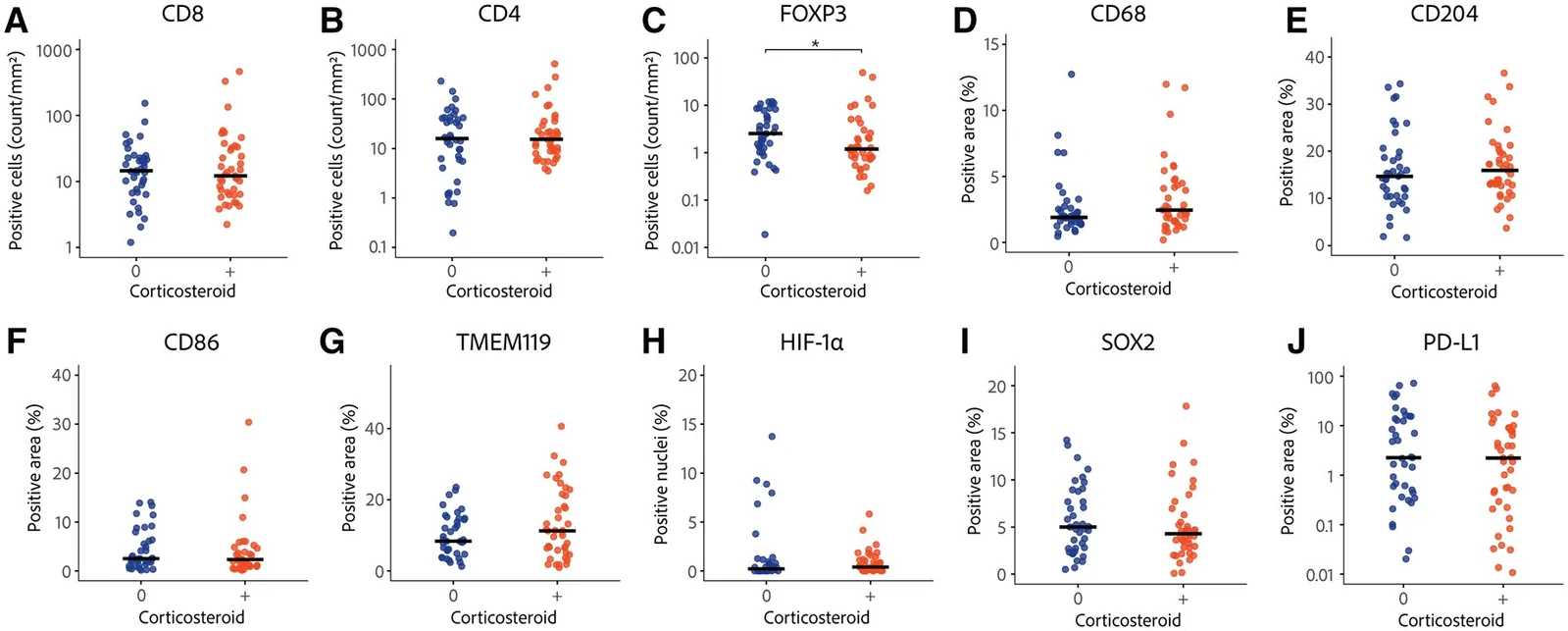
Immunohistochemical marker abundance according to preoperative corticosteroid exposure. (A) CD8; (B) CD4; (C) FOXP3; (D) CD68; (E) CD204; (F) CD86; (G) TMEM119; (H) HIF-1α; (I) SOX2; (J) PD-L1. Individual points represent patient samples, and lines indicate medians. Statistical significance is denoted with an asterisk where *P* < .05. Y-axes in A, B, C, and J have been log-transformed. Analyses included 39 non-treated and 41 corticosteroid-treated patients, except for CD86, which included 39 non-treated and 36 corticosteroid-treated patients.

We then subdivided the corticosteroid-treated patients by daily dose to explore dose-related patterns (Figure 4A-J, Table S3). FOXP3+ regulatory T cells were lowest in patients receiving 12.5-75 mg/day corticosteroids (overall *P* = .030; 0 mg vs 12.5-75 mg: *P* = .031). In contrast, the highest corticosteroid dose was associated with increased abundance of the myeloid/macrophage markers CD68 and CD204 (CD68: overall *P* = .015; 0 mg vs >75 mg: *P* = .037; 12.5-75 mg vs >75 mg: *P* = .046; CD204: overall *P* = .022; 12.5-75 mg vs >75 mg: *P* = .023; Figure 4 and Table S3). No consistent dose-related differences were observed for CD8, CD4, CD86, TMEM119, HIF-1α, SOX2, or PD-L1.

**Figure 4.**
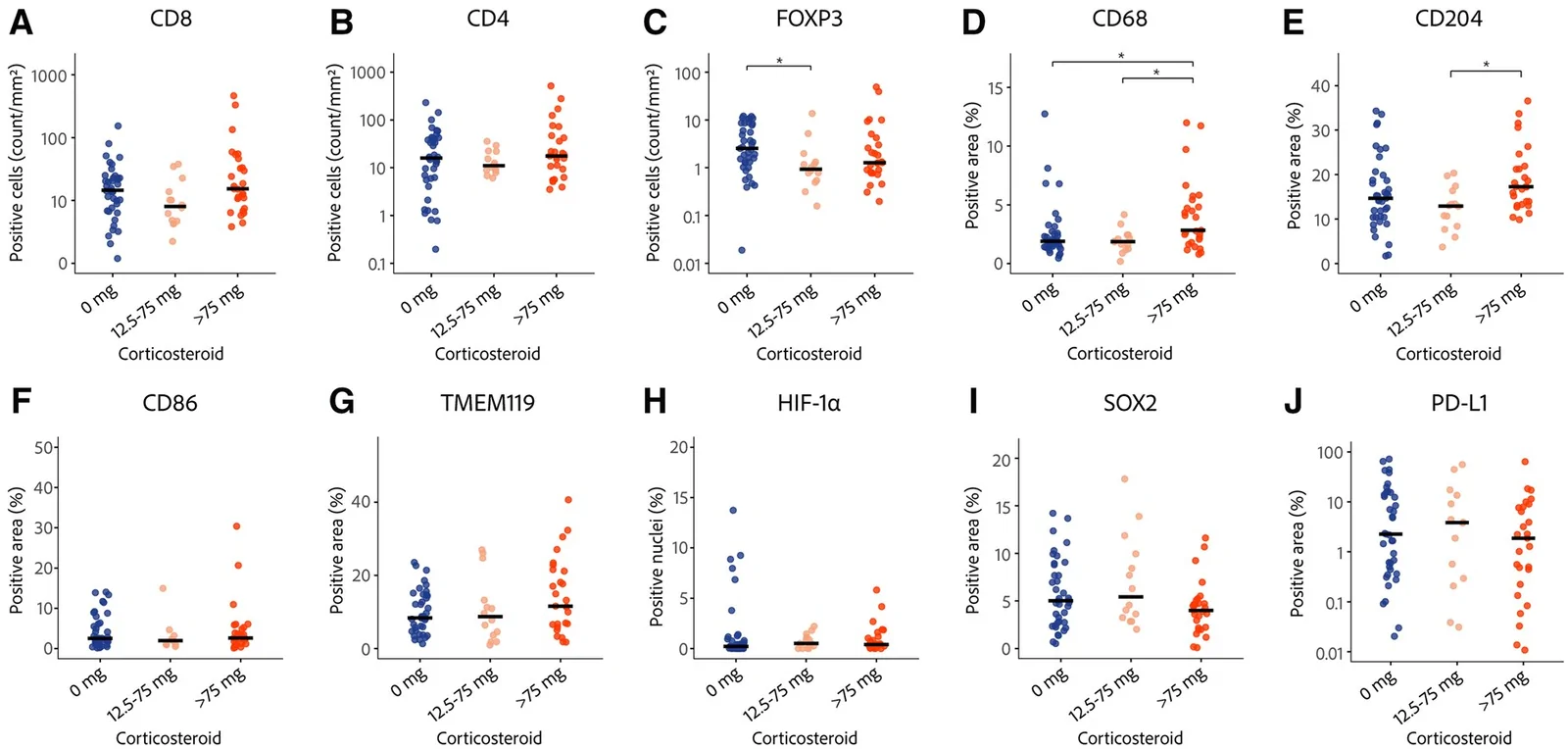
Immunohistochemical marker abundance according to preoperative corticosteroid dose. (A) CD8; (B) CD4; (C) FOXP3; (D) CD68; (E) CD204; (F) CD86; (G) TMEM119; (H) HIF-1α; (I) SOX2; (J) PD-L1. Individual points represent patient samples, and lines indicate medians. Statistical significance is denoted with an asterisk where *P* < .05. Y-axes in A, B, C, and J have been log-transformed. Dose subgroup analyses included 39 patients receiving 0 mg, 14 receiving 12.5-75 mg, and 27 receiving >75 mg corticosteroids, except for CD86, which included 39, 11, and 25 patients in the respective groups.

When assessing correlations across the marker panel, T-cell and myeloid/macrophage-associated markers were broadly positively correlated, particularly CD4, CD8, FOXP3, CD68, CD204, and CD86, consistent with coordinated variation in immune-cell infiltration across tumors. TMEM119 also showed positive correlations with several myeloid/macrophage-associated markers. PD-L1 showed modest positive correlations with CD8, CD4, and CD204, whereas HIF-1α and SOX2 did not show strong positive correlations with this immune-marker cluster (Figure S1C).

### Larger Tumor Size Does Not Explain Corticosteroid-Associated Immune Changes

MRI-based tumor size was larger in corticosteroid-treated patients than in non-treated patients (*P* < .001; Table 1 and Figure S1A and B). We therefore asked whether larger tumor size could explain the corticosteroid-associated immune-marker changes. These analyses were performed in the subset of patients with available MRI-based tumor-size measurements (*n* = 51).

First, we examined whether tumor size itself was associated with the three main corticosteroid-associated markers. Spearman correlation analysis showed no significant association between tumor size and FOXP3, CD68, or CD204 abundance (Figure S1D-F). We then performed tumor-size-adjusted models for the three main corticosteroid-associated markers (Table S4). Corticosteroid exposure remained associated with lower FOXP3 abundance after adjustment for tumor size. For CD68 and CD204, which were primarily altered in the dose-subgroup analyses, the high-dose-associated pattern remained evident after adjustment, with higher CD68 and CD204 abundance in the >75 mg group than in the 12.5-75 mg group.

As larger tumors may contain more necrotic or hypoxic regions that could influence immune-marker expression, we also quantified HIF-1α as a hypoxia-related marker. Although areas containing extensive necrosis were excluded from image analysis, smaller necrotic foci and surrounding pseudopalisading tumor cells were included when present. HIF-1α expression did not differ significantly according to corticosteroid exposure or dose group (Figures 3 and 4; Table S3). Together, these analyses suggest that the observed immune-marker patterns were not simply explained by larger tumor size or broad group-level differences in this hypoxia-related marker, although the dose-related CD68 and CD204 findings should be interpreted with caution.

### Comparison of Overall Survival Across Treatment Groups

When comparing overall survival using the Mantel-Cox log-rank test, no significant differences were observed in either the 2-group comparison between non-treated and corticosteroid-treated patients or the 3-group comparison between patients receiving 0 mg, 12.5-75 mg, and >75 mg corticosteroids (Figure S2A and B).

## Discussion

Despite the widespread use of corticosteroids in GBM treatment, their effects on the TME remain poorly characterized. In this study, preoperative corticosteroid exposure was associated with a marked reduction in FOXP3+ cells, while dose-subgroup analyses showed a high-dose-associated increase in CD68 and CD204 myeloid/macrophage-marker abundance. In contrast, CD4, CD8, CD86, TMEM119, HIF-1α, SOX2, and PD-L1 did not show consistent differences according to corticosteroid exposure or dose. Together, these findings suggest that corticosteroid-associated changes within the GBM immune microenvironment are selective rather than reflecting a uniform reduction or expansion of immune-cell populations.

FOXP3+ regulatory T cells are important contributors to the immunosuppressive milieu of GBM and have been linked to impaired anti-tumor immunity and poor overall survival.^27^^,^^28^ We observed a marked reduction in FOXP3+ cells in corticosteroid-treated tumors, consistent with lower intratumoral Treg abundance in association with corticosteroid exposure. Previous clinical studies in GBM patients, based on flow cytometric immunophenotyping of peripheral blood, have reported reduced absolute lymphocyte counts, including Tregs, alongside expansion of the myeloid compartment in peripheral blood in response to corticosteroids.^6^^,^^7^ While these studies primarily reflect systemic immune effects, their findings are consistent with the reduced FOXP3+ Treg infiltration observed in the tumor tissue in our cohort. However, FOXP3 is not entirely specific to regulatory T cells in humans, as activated conventional T cells can transiently express FOXP3 without acquiring a regulatory phenotype.^29^ This is particularly relevant to single-marker immunohistochemistry, which cannot definitively identify Tregs in human brain tumors.^30^ However, a GBM study using dual immunohistochemical staining found strong concordance between FOXP3 expression alone and CD4+FOXP3+ cells, supporting its use as a marker of Treg abundance in this setting.^28^ We therefore consider the observed reduction in FOXP3+ cells most consistent with reduced Treg abundance, while acknowledging that our single-marker analysis cannot definitively establish the phenotype of every FOXP3+ cell.

In contrast, we observed no significant differences in CD4+ or CD8+ T cell densities. This discrepancy compared with systemic immune studies may reflect the already profoundly immunosuppressed nature of the GBM TME, which may limit further detectable reductions in lymphoid populations. Moreover, systemic immune alterations do not necessarily translate to corresponding changes within tumor tissue, as previously demonstrated in smaller patient cohorts.^11^ However, unchanged CD4+ and CD8+ T cell densities do not exclude corticosteroid-associated changes in T cell activation or functional state, which were not assessed in the present study.

When comparing patients not receiving corticosteroids with those treated at different dosage levels, we found a higher CD68+ area in patients receiving >75 mg/day corticosteroids compared with both non-treated patients and those receiving 12.5-75 mg/day. CD68 is associated with high-grade disease, immunosuppression, and adverse prognosis in GBM.^31^^,^^32^ While CD68 expression has been studied in glioma tissue,^11^ this is, to our knowledge, the first study to identify a possible association between corticosteroid exposure and increased CD68 marker abundance in human GBM tissue. CD204 showed a related high-dose-associated pattern, with higher abundance in the high- versus low-to-moderate-dose group, whereas the difference from non-treated patients was not significant. Notably, the untreated and low-to-moderate dosage groups included tumors with CD204+ area fractions in the 1.7%-9.4% range, whereas no tumors in the high-dosage group fell within this range, illustrating the different CD204 distribution in the high-dose group. Because these findings arose from dose-stratified analyses with relatively small subgroups, their strength and generalizability remain uncertain, and they should not be interpreted as evidence of a definitive dose-response relationship. Consistent with this, a recent single-cell RNA-seq analysis of 37 *IDH*-wildtype gliomas showed that high-dose dexamethasone treatment (>8 mg/day) was associated with enrichment of an immunosuppressive myeloid program, which increased with escalating dexamethasone dose.^33^ Although the dexamethasone-associated program was distinct from the scavenger immunosuppressive program containing *MSR1* (CD204) and *MRC1* (CD206), these data support the biological plausibility of corticosteroid-associated modulation of myeloid programs. As demonstrated in some of our previous studies by multivariate analysis, CD204 is associated with poor prognosis.^34-36^ Together, these findings suggest that high-dose corticosteroid exposure may be linked to myeloid-marker changes with potential clinical relevance, although further studies are needed to validate this association.

To further characterize the myeloid compartment, we also assessed CD86 and TMEM119. Neither marker differed significantly according to corticosteroid exposure or dose. Although CD86 has traditionally been associated with an M1-like phenotype, we do not interpret CD86 and CD204 as a direct measure of M1/M2 balance because this binary framework does not adequately capture the heterogeneity and plasticity of TAMs in human GBM.^37^ TMEM119 is a microglia-associated marker that can help distinguish microglia from peripheral monocytes/macrophages in glioma, although its expression can change under pathological conditions.^38^ Thus, the unchanged TMEM119 signal does not exclude changes in the relative contributions or functional states of resident microglia and monocyte-derived macrophages, which cannot be definitively resolved by single-marker immunohistochemistry.

In the present study, we found no significant difference in overall PD-L1 abundance between corticosteroid-treated and untreated human GBM tissue. This finding aligns with a clinical immunohistochemical study in non-small cell lung cancer tissue, which likewise reported no association between prednisolone use and PD-L1 expression.^26^ Preclinical studies have reported divergent effects of corticosteroids on PD-L1 expression across GBM and other cancer models,^23-25^ suggesting that these effects are context dependent. Indeed, our use of single-staining immunohistochemistry limits our ability to distinguish between PD-L1–expressing cell types. Because our dose-subgroup analyses showed higher CD68 marker abundance in patients receiving the highest corticosteroid doses, and CD68+ tumor-associated macrophages are a frequent cellular source of PD-L1 across several cancers,^39-41^ a potential increase in PD-L1-expressing macrophages could be masked by changes in another cellular compartment. Accordingly, similar overall PD-L1 abundance between treatment groups may represent different underlying cellular compositions of PD-L1 expression. Thus, unchanged overall PD-L1 abundance does not exclude corticosteroid-associated changes in its cellular distribution or regulation.

In clinical studies, preoperative corticosteroid use is often insufficiently accounted for and has been associated with poorer survival in patients receiving immunotherapy, although its role as an independent prognostic factor remains unclear.^42^^,^^43^ Prednisolone use at both first-line treatment and recurrence has, however, been identified as an independent predictor of poor survival in selected cohorts.^44^^,^^45^ In the present cohort, we observed no significant difference in overall survival according to corticosteroid exposure or dose, but survival was not the primary endpoint, and our analysis was not designed to determine whether corticosteroid exposure independently affects prognosis.

The present study benefits from a well-characterized and balanced patient cohort and represents the largest analysis to date of corticosteroid-associated immune alterations in human GBM tissue. In contrast, previous studies were limited by small cohorts and reliance on tissue microarrays,^11^ which sample only a limited fraction of the tumor and may therefore incompletely capture intratumoral immune heterogeneity.^11^^,^^36^

A relevant limitation is that corticosteroid-treated patients had significantly larger tumors, which may represent a potential confounder despite identical histological selection criteria. After adjustment for tumor size, corticosteroid exposure remained significantly associated with lower FOXP3 abundance, while the higher abundance of CD68 and CD204 in the >75 mg/day group compared with the 12.5-75 mg/day group also remained statistically significant. These analyses were restricted to the 51 patients with available MRI-based measurements, and some confounding by tumor size therefore cannot be excluded. HIF-1α is characteristically expressed in hypoxic GBM regions, including pseudopalisading tumor cells surrounding necrosis,^46^ and we observed this characteristic staining pattern in our samples. However, overall HIF-1α abundance did not differ according to corticosteroid exposure or dose, arguing against a broad group-level difference in hypoxia within the analyzed tumor tissue. Because extensively necrotic areas were not included, this analysis does not provide a measure of total necrotic burden. More broadly, as a retrospective observational study, corticosteroid treatment was determined by clinical indication rather than randomly assigned, and residual confounding by factors such as edema, neurological symptoms, treatment duration, and timing relative to surgery cannot be excluded. The observed differences should therefore be interpreted as associations rather than evidence of direct corticosteroid effects.

Future prospective studies with more detailed characterization of corticosteroid indication and exposure are needed to validate our findings. Given the pronounced T cell exhaustion phenotype in GBM,^47^ analyses should extend beyond PD-L1 and include more detailed characterization of T cell activation and myeloid cell states, ideally using spatial or multiplex approaches. Such approaches could directly assess CD4 and FOXP3 co-expression, distinguish resident microglia from monocyte-derived macrophages, characterize myeloid states beyond the M1/M2 framework, and identify the cellular sources of PD-L1 expression.

## Conclusion

Our study shows that preoperative corticosteroid exposure in GBM is associated with a reduction in FOXP3+ regulatory T cells and a high-dose-associated increase in CD68 and CD204 myeloid/macrophage-marker abundance. These findings suggest that corticosteroid use is associated with changes in key immune populations within the TME and highlight the importance of considering corticosteroid exposure when interpreting immune profiles in GBM. Further studies are needed to define the underlying cellular mechanisms and determine how these changes may influence responses to immune-based therapies.

## Supplementary Material

vdag258_Supplementary_Data

## Data Availability

The data generated and analyzed during this study are available from the corresponding author upon reasonable request.
