# Supplementary material for: Corticosteroid treatment modulates immune cell populations in the glioblastoma microenvironment
Source: Neurooncol Adv. 2026 Oct 7;8(1):vdag258. doi: 10.1093/noajnl/vdag258 (PMC13722118; doi:10.1093/noajnl/vdag258)
Supplement: Supplementary file 1 [file vdag258_supplementary_data.docx]

Supplementary materials

**Table S1. Antibodies and protocols used for immunohistochemistry.**

| **Antibody** | **Vendor, clone, and catalog no.** | **Host species, clonality** | **Antigen retrieval (buffer, min/°C)** | **Dilution** | **Ancillaries** | **Primary antibody incubation time** |
| --- | --- | --- | --- | --- | --- | --- |
| **CD8** | Dako, C8/144B | Ms, mAb | CC1, 48 min/100 °C | 1:50 | None | 32 min |
| **CD4** | Roche, SP35 | Rb, mAb | CC2, 32 min/100 °C | RTU | None | 4 min |
| **FOXP3** | Invitrogen, 236A/E7 | Ms, mAb | CC1, 48 min/100 °C | 1:50 | Discovery Amplification HQ-HRP 12 min. | 20 min |
| **CD68** | Roche, KP1 | Ms, mAb | CC2, 40 min/100 °C | RTU | None | 32 min |
| **CD204** | Cosmo Bio Co., Ltd., SRA-E5 | Ms, mAb | CC1, 32 min/100 °C | 1:600 | Discovery Amplification HQ-HRP 20 min. | 32 min |
| **CD86** | R&D Systems, AF-141-NA | Gt, pAb | CC1, 54 min/100 °C | 1:100 | None | 60 min |
| **TMEM119** | Abcam, ab185333 | Rb, pAb | CC1, 32 min/100 °C | 1:50 | None | 60 min |
| **SOX2** | Invitrogen/ eBioscience™, Btjce, 14-9811-82 | Rt, mAb | CC2, 48 min/100 °C | 1:800 | None | 32 min |
| **HIF-1α** | BD Transduction Laboratories™, 54/HIF-1α | Ms, mAb | CC1, 48 min/100 °C | 1:100 | Discovery Amplification HQ-HRP 8 min. | 32 min |
| **PD-L1** | Agilent, 22C3 | Ms, mAb | CC1, 64 min/95 °C | 1:50 | Discovery Amplification HQ-HRP 12 min. | 16 h |

**Notes**

All immunohistochemical staining was performed on the Ventana Discovery Ultra platform (Ventana Medical Systems).

Abbreviations: Ms, mouse; Rb, rabbit; Rt, rat; Gt, goat; mAb, monoclonal antibody; pAb, polyclonal antibody; RTU, ready to use; CC1/CC2, antigen retrieval buffers.

**Table S2. Overview of antibodies used in the study and their corresponding purpose.**

| **Antibody** | **Purpose** |
| --- | --- |
| **CD8** | Glycoprotein functioning as a co-receptor for the T-cell receptor and interacting with antigen–MHC I complexes, predominantly expressed by CD8+ T cells^1^. In this study, CD8 was used to assess CD8+ T-cell abundance. |
| **CD4** | Glycoprotein functioning as a co-receptor for the T-cell receptor and interacting with antigen–MHC II complexes, expressed by CD4+ T cells and several other immune-cell populations^2^. In this study, CD4 was used to assess CD4+ T-cell abundance. |
| **FOXP3** | Transcription factor strongly associated with regulatory T cells, although activated conventional T cells can also express FOXP3 without acquiring a regulatory phenotype^3–5^. In this study, FOXP3 was used as a marker of regulatory T-cell abundance. |
| **CD68** | Myeloid/macrophage-associated protein widely used to identify tumor-associated microglia/macrophages in glioma^6,7^. In this study, CD68 was used as a marker of myeloid/macrophage abundance. |
| **CD204** | Scavenger receptor expressed by a subset of tumor-associated microglia/macrophages and associated with immunosuppressive myeloid phenotypes and adverse prognosis in glioma^8–10^. In this study, CD204 was used as a myeloid/macrophage-associated marker. |
| **CD86** | Co-stimulatory molecule expressed by antigen-presenting cells and commonly used as an activation-associated myeloid marker. Although CD86 has traditionally been associated with an M1-like phenotype, macrophage states in human GBM are more heterogeneous than the binary M1/M2 framework suggests^11^. In this study, CD86 was used as an activation-associated myeloid marker. |
| **TMEM119** | Microglia-associated transmembrane protein that can help distinguish resident microglia from peripheral monocytes/macrophages, although its expression may change under pathological conditions^12^. In this study, TMEM119 was used to assess microglia-associated signal. |
| **SOX2** | Transcription factor associated with stem-like and undifferentiated tumor-cell states in glioblastoma^13^. In this study, SOX2 was used as a tumor-cell-associated marker. |
| **HIF-1α** | Transcription factor stabilized under hypoxic conditions and characteristically expressed in hypoxic GBM regions, including pseudopalisading tumor cells surrounding necrosis^14^. In this study, HIF-1α was used as a hypoxia-related marker. |
| **PD-L1** | Transmembrane immune-checkpoint ligand expressed by multiple cell types, including tumor and immune cells. Binding of PD-L1 to PD-1 inhibits T-cell activation and effector function^15–19^. In this study, PD-L1 was used to assess overall immune-checkpoint expression within the analyzed tumor regions. |

**References**

1. Raskov H, Orhan A, Christensen JP, Gögenur I. Cytotoxic CD8+ T cells in cancer and cancer immunotherapy. Br J Cancer. 2021;124(2):359-367. doi:10.1038/s41416-020-01048-4
2. Luckheeram RV, Zhou R, Verma AD, Xia B. CD4+T cells: differentiation and functions. Clin Dev Immunol. 2012;2012:925135. doi:10.1155/2012/925135
3. Sayour EJ, McLendon P, McLendon R, et al. Increased proportion of FoxP3+ regulatory T cells in tumor infiltrating lymphocytes is associated with tumor recurrence and reduced survival in patients with glioblastoma. Cancer Immunol Immunother CII. 2015;64(4):419-427. doi:10.1007/s00262-014-1651-7
4. Allan SE, Crome SQ, Crellin NK, et al. Activation-induced FOXP3 in human T effector cells does not suppress proliferation or cytokine production. Int Immunol. 2007;19(4):345-354. doi:10.1093/intimm/dxm014
5. Jacobs JFM, Idema AJ, Bol KF, et al. Prognostic significance and mechanism of Treg infiltration in human brain tumors. J Neuroimmunol. 2010;225(1-2):195-199. doi:10.1016/j.jneuroim.2010.05.020
6. Zhang J, Li S, Liu F, Yang K. Role of CD68 in tumor immunity and prognosis prediction in pan-cancer. Sci Rep. 2022;12(1):7844. doi:10.1038/s41598-022-11503-2
7. Wang L, Zhang C, Zhang Z, et al. Specific clinical and immune features of CD68 in glioma via 1,024 samples. Cancer Manag Res. 2018;10:6409-6419. doi:10.2147/CMAR.S183293
8. Yuan Y, Zhao Q, Zhao S, et al. Characterization of transcriptome profile and clinical features of a novel immunotherapy target CD204 in diffuse glioma. Cancer Med. 2019;8(8):3811-3821. doi:10.1002/cam4.2312
9. Sørensen MD, Kristensen BW. Tumour-associated CD204+ microglia/macrophages accumulate in perivascular and perinecrotic niches and correlate with an interleukin-6-enriched inflammatory profile in glioblastoma. Neuropathol Appl Neurobiol. 2022;48(2):e12772. doi:10.1111/nan.12772
10. Sørensen MD, Dahlrot RH, Boldt HB, Hansen S, Kristensen BW. Tumour-associated microglia/macrophages predict poor prognosis in high-grade gliomas and correlate with an aggressive tumour subtype. Neuropathol Appl Neurobiol. 2018;44(2):185-206. doi:10.1111/nan.12428
11. Khan F, Pang L, Dunterman M, Lesniak MS, Heimberger AB, Chen P. Macrophages and microglia in glioblastoma: heterogeneity, plasticity, and therapy. J Clin Invest. 2023;133(1):e163446. doi:10.1172/JCI163446
12. Haage V, Semtner M, Vidal RO, et al. Comprehensive gene expression meta-analysis identifies signature genes that distinguish microglia from peripheral monocytes/macrophages in health and glioma. Acta Neuropathol Commun. 2019;7(1):20. doi:10.1186/s40478-019-0665-y
13. Garros-Regulez L, Garcia I, Carrasco-Garcia E, et al. Targeting SOX2 as a Therapeutic Strategy in Glioblastoma. Front Oncol. 2016;6:222. doi:10.3389/fonc.2016.00222
14. Brat DJ, Castellano-Sanchez AA, Hunter SB, et al. Pseudopalisades in glioblastoma are hypoxic, express extracellular matrix proteases, and are formed by an actively migrating cell population. Cancer Res. 2004;64(3):920-927. doi:10.1158/0008-5472.can-03-2073
15. Ribas A. Adaptive Immune Resistance: How Cancer Protects from Immune Attack. Cancer Discov. 2015;5(9):915-919. doi:10.1158/2159-8290.CD-15-0563
16. Nduom EK, Wei J, Yaghi NK, et al. PD-L1 expression and prognostic impact in glioblastoma. Neuro-Oncol. 2016;18(2):195-205. doi:10.1093/neuonc/nov172
17. Gabrusiewicz K, Li X, Wei J, et al. Glioblastoma stem cell-derived exosomes induce M2 macrophages and PD-L1 expression on human monocytes. OncoImmunology. 2018;7(4):e1412909. doi:10.1080/2162402X.2017.1412909
18. Keir ME, Butte MJ, Freeman GJ, Sharpe AH. PD-1 and Its Ligands in Tolerance and Immunity. Annu Rev Immunol. 2008;26:677-704. doi:10.1146/annurev.immunol.26.021607.090331
19. Freeman GJ, Long AJ, Iwai Y, et al. Engagement of the Pd-1 Immunoinhibitory Receptor by a Novel B7 Family Member Leads to Negative Regulation of Lymphocyte Activation. J Exp Med. 2000;192(7):1027-1034. doi:10.1084/jem.192.7.1027

**Table S3. IHC marker quantification according to corticosteroid treatment and dose subgroup**

**A. Treatment group comparison**

| **Marker** | **Measurement** | **Non-treated** | **Steroid-treated** | **P value** |
| --- | --- | --- | --- | --- |
| CD8 (median) | Positive cells (count/mm2) | 14.55 | 12.22 | 0.962 |
| CD4 (median) | Positive cells (count/mm2) | 15.89 | 15.30 | 0.781 |
| FOXP3 (median) | Positive cells (count/mm2) | 2.53 | 1.19 | **0.021*** |
| CD68 (median) | Positive area (%) | 1.89 | 2.44 | 0.115 |
| CD204 (median) | Positive area (%) | 14.67 | 15.92 | 0.455 |
| CD86 (median) | Positive area (%) | 2.53 | 2.35 | 0.530 |
| TMEM119 (median) | Positive area (%) | 8.37 | 11.29 | 0.323 |
| HIF-1α (median) | Positive nuclei (%) | 0.21 | 0.42 | 0.179 |
| SOX2 (median) | Positive area (%) | 5.01 | 4.29 | 0.421 |
| PD-L1 (median) | Positive area (%) | 2.26 | 2.22 | 0.362 |

**B. Steroid dose subgroup comparison**

| **Marker** | **Measurement** | **0 mg** | **12.5–75 mg** | **>75 mg** | **Overall P value** | **0 vs 12.5–75 mg** | **0 vs >75 mg** | **12.5–75 vs >75 mg** |
| --- | --- | --- | --- | --- | --- | --- | --- | --- |
| CD8 (median) | Positive cells (count/mm2) | 14.55 | 8.05 | 15.33 | 0.069 | 0.298 | 0.965 | 0.062 |
| CD4 (median) | Positive cells (count/mm2) | 15.89 | 11.03 | 17.59 | 0.472 | >0.99 | >0.99 | 0.700 |
| FOXP3 (median) | Positive cells (count/mm2) | 2.53 | 0.93 | 1.27 | **0.030*** | **0.031*** | 0.423 | 0.574 |
| CD68 (median) | Positive area (%) | 1.89 | 1.87 | 2.83 | **0.015*** | >0.99 | **0.037*** | **0.046*** |
| CD204 (median) | Positive area (%) | 14.67 | 12.91 | 17.25 | **0.022*** | 0.570 | 0.184 | **0.023*** |
| CD86 (median) | Positive area (%) | 2.53 | 1.97 | 2.61 | 0.508 | 0.753 | >0.99 | >0.99 |
| TMEM119 (median) | Positive area (%) | 8.37 | 8.72 | 11.59 | 0.226 | >0.99 | 0.384 | 0.477 |
| HIF-1α (median) | Positive nuclei (%) | 0.21 | 0.51 | 0.40 | 0.398 | >0.99 | 0.624 | >0.99 |
| SOX2 (median) | Positive area (%) | 5.01 | 5.43 | 4.01 | 0.129 | >0.99 | 0.356 | 0.191 |
| PD-L1 (median) | Positive area (%) | 2.26 | 3.84 | 1.87 | 0.491 | >0.99 | 0.735 | >0.99 |

**Notes**

Unless otherwise indicated, analyses included 39 non-treated and 41 corticosteroid-treated patients. Dose subgroup analyses included 39 patients receiving 0 mg, 14 receiving 12.5–75 mg, and 27 receiving >75 mg corticosteroids. For CD86, analyses included 39 non-treated and 36 corticosteroid-treated patients, with dose subgroup sizes of 39, 11, and 25, respectively. Marker values are shown as medians. Two-group comparisons used the Wilcoxon rank-sum test, and dose-group comparisons used the Kruskal–Wallis test followed by Dunn’s multiple comparisons test with Bonferroni correction. P values are reported to three decimal places, with adjusted P values >0.99 reported as >0.99. Significant values are indicated by an asterisk (*P* < 0.05).

**Table S4. Tumor-size-adjusted sensitivity analyses for key corticosteroid-associated markers**

**A. Binary corticosteroid exposure model**

| **Marker** | **Comparison** | **N total** | **Adjusted rank difference (95% CI)** | **P value** |
| --- | --- | --- | --- | --- |
| FOXP3 | Any corticosteroid vs 0 mg | 51 | -11.4 (-20.3 to -2.4) | **0.014*** |
| CD68 | Any corticosteroid vs 0 mg | 51 | 2.2 (-7.3 to 11.8) | 0.637 |
| CD204 | Any corticosteroid vs 0 mg | 51 | 2.6 (-6.6 to 11.8) | 0.569 |

**B. Corticosteroid dose-group model**

| **Marker** | **Comparison** | **N total** | **N 0 mg** | **N 12.5-75 mg** | **N >75 mg** | **Adjusted rank difference (95% CI)** | **P value** |
| --- | --- | --- | --- | --- | --- | --- | --- |
| FOXP3 | 12.5-75 mg vs 0 mg | 51 | 21 | 10 | 20 | -14.7 (-26.1 to -3.2) | **0.013*** |
| FOXP3 | >75 mg vs 0 mg | 51 | 21 | 10 | 20 | -9.6 (-19.4 to 0.2) | 0.056 |
| FOXP3 | >75 mg vs 12.5-75 mg | 51 | 21 | 10 | 20 | 5.1 (-6.0 to 16.2) | 0.361 |
| CD68 | 12.5-75 mg vs 0 mg | 51 | 21 | 10 | 20 | -5.3 (-17.1 to 6.5) | 0.370 |
| CD68 | >75 mg vs 0 mg | 51 | 21 | 10 | 20 | 6.4 (-3.7 to 16.5) | 0.206 |
| CD68 | >75 mg vs 12.5-75 mg | 51 | 21 | 10 | 20 | 11.7 (0.3 to 23.2) | **0.045*** |
| CD204 | 12.5-75 mg vs 0 mg | 51 | 21 | 10 | 20 | -6.3 (-17.4 to 4.9) | 0.263 |
| CD204 | >75 mg vs 0 mg | 51 | 21 | 10 | 20 | 7.6 (-2.0 to 17.1) | 0.117 |
| CD204 | >75 mg vs 12.5-75 mg | 51 | 21 | 10 | 20 | 13.8 (3.0 to 24.7) | **0.013*** |

**Notes**

Models were restricted to patients with available MRI-based tumor-size measurements. The outcome is marker rank. Tumor size was included as tumor area per 1000 mm². In section A, the adjusted rank difference compares patients receiving any preoperative corticosteroid treatment with non-treated patients. In section B, positive adjusted rank differences indicate higher marker rank in the first dose group listed in the comparison. *P* values are rounded to three decimal places. Significant *P* values are indicated with an asterisk (**P* < 0.05).

**FIGURE S1**

**Supplementary Figure 1: MRI-based tumor size and correlations between immunohistochemical markers.**

(A-B) MRI-based tumor size according to preoperative corticosteroid exposure (A) and corticosteroid dose group (B). Lines indicate medians. Statistical significance is denoted with asterisks, where ** = *P* < 0.01 and *** = *P* < 0.001.

(C) Spearman correlation heatmap showing pairwise relationships between the immunohistochemical markers HIF-1α, SOX2, FOXP3, CD8, CD68, CD204, CD4, CD86, TMEM119, and PD-L1. The color scale indicates the direction and magnitude of the Spearman correlation coefficient (ρ). Asterisks indicate statistical significance, where P < 0.05, P < 0.01, P < 0.001, and P < 0.0001.

(D–F) Spearman correlation analyses of MRI-based tumor size with FOXP3 (D), CD68 (E), and CD204 (F) abundance in patients with available tumor-size measurements (n = 51). Points are colored according to preoperative corticosteroid exposure.

**FIGURE S2**

**Supplementary Figure 2: Overall survival according to preoperative corticosteroid exposure and dose.**

A) Kaplan–Meier analysis comparing overall survival between patients who did and did not receive preoperative corticosteroids. B) Kaplan–Meier analysis comparing overall survival between patients receiving 0 mg, 12.5–75 mg, and >75 mg corticosteroids. Dashed vertical lines indicate median overall survival. *P* values were determined using the Mantel–Cox log-rank test.
